# Trends in Inhibitors, Structural Modifications, and Structure–Function Relationships of Phosphodiesterase 4: A Review

**DOI:** 10.3390/biom16010079

**Published:** 2026-01-03

**Authors:** Antonio Sánchez-Belmonte, Adrián Matencio, Irene Conesa, Francisco José Vidal-Sánchez, Francesco Trotta, José Manuel López-Nicolás

**Affiliations:** 1 Departamento de Bioquímica y Biología Molecular A, Unidad Docente de Biología, Facultad de Veterinaria, Regional Campus of International Excellence “Campus Mare Nostrum”, Universidad de Murcia, 30100 Murcia, Spain; antonio.s.b@um.es (A.S.-B.); irene.conesav@um.es (I.C.); fjose.vidal@um.es (F.J.V.-S.); josemln@um.es (J.M.L.-N.); 2 Department of Chemistry, University of Turin, Via Pietro Giuria 7, 10125 Torino, Italy; francesco.trotta@unito.it

**Keywords:** PDE4, cAMP, structural biology, natural inhibitors, rational drug design, selectivity

## Abstract

Phosphodiesterase 4 (PDE4) is a key enzyme responsible for the hydrolysis of cyclic adenosine monophosphate (cAMP), thereby regulating essential signaling pathways involved in inflammation and immune modulation. Structural studies have demonstrated a high degree of conservation within the catalytic domains of PDE4 isoforms, accompanied by subtle conformational variations that underlie their selectivity and tissue-specific distribution. Elucidating these structural features has been instrumental in guiding the rational design of PDE4 inhibitors. Although synthetic PDE4 inhibitors such as roflumilast and apremilast exhibit significant therapeutic efficacy, their clinical application is often limited by dose-dependent adverse effects. These effects primarily arise from insufficient isoform selectivity, as current inhibitors tend to target multiple PDE4 subtypes indiscriminately, resulting in off-target pharmacological actions and reduced tolerability. In contrast, natural products—including flavonoids, terpenoids, and related polyphenolic compounds such as curcumin, α-mangostin, and their derivatives—have emerged as promising molecular scaffolds. Their lower toxicity, favorable biocompatibility, and structural diversity enable fine-tuning of potency and selectivity through rational modification. Integrating structural insights derived from crystallographic and computational studies with the optimization of natural compounds offers a sustainable and effective strategy for the development of safer, isoform-selective PDE4-targeted therapies.

## 1. Introduction

Inflammation constitutes a complex and highly regulated biological response of the immune system to harmful stimuli, such as pathogens, damaged cells, or cytotoxic compounds. This process plays a central role in numerous diseases, including cancer and various autoimmune disorders such as psoriasis and rheumatoid arthritis [1,2]. During the inflammatory response, different immune mediators, immune cells, and blood vessels act in a coordinated manner with the purpose of repairing tissue damage, eliminating the causal agent, and ultimately restoring homeostasis and promoting regeneration processes [3]. This process can be altered by various genetic and environmental factors that affect the resolution of inflammation. An acute and uncontrolled inflammatory response can lead to chronic inflammation, which in turn can result in chronic inflammatory disease [4].

The study of inflammation has focused on pharmacological strategies, including both drugs and bioactive compounds, to modulate inflammatory responses. However, current drugs present a large number of adverse side effects and general toxicity due to their lack of selectivity and affinity for the enzymatic targets involved in this process. Therefore, studying these molecular targets to improve the pharmacological profile of anti-inflammatory therapies represents a key step forward [5].

Phosphodiesterase 4 (PDE4) is a key subfamily of metalloenzymes that regulate the hydrolysis of cyclic adenosine monophosphate (cAMP) to adenosine monophosphate (AMP). These hydrolase enzymes are found in epithelial, neuronal, and immune cells, where they play a fundamental role in the regulation of inflammation [6]. Currently, compounds such as roflumilast, crisaborole, and difamilast are used to inhibit PDE4 in inflammatory contexts, approved or in advanced clinical phases [7]. Nevertheless, simultaneous inhibition of other phosphodiesterase families has led to undesirable side effects. This situation has prompted extensive investigation into the structural differences between the PDE4 subfamilies, which, despite sharing a highly conserved catalytic domain, exhibit subtle variations in amino acid sequence [8].

In this context, rational drug design emerges as an optimal strategy to achieve more selective inhibition. However, the structural complexity of the enzyme hinders de novo design, highlighting the relevance of using natural bioactive compounds as starting points. Many of these compounds possess chemical structures analogous to those of the endogenous enzyme substrate, offering a promising structural foundation for the development of new selective inhibitors.

“Unlike previous reviews, this work provides a comprehensive and focused analysis of natural PDE4 inhibitors, a particularly relevant and emerging area in the search for safer and more selective PDE4-targeted therapies. In addition, it systematically integrates the structural differences among PDE4 isoforms, which represent one of the most promising strategies for achieving isoform selectivity. This analysis is presented within a complete overview of PDE4 biology, including its enzymatic function, structural organization, and involvement in inflammatory diseases, thereby offering a unified perspective that bridges natural product chemistry and structure-guided isoform-selective inhibitor design.”

## 2. Inflammation

### 2.1. Basic Principles

The inflammatory response is triggered once Toll-like receptors (TLRs) and other pattern recognition receptors (PRRs) detect tissue damage or pathogenic signals. When this occurs, epithelial cells and resident macrophages within tissues initiate the inflammatory cascade [9,10,11,12,13]. These cells produce various proinflammatory cytokines and chemokines, such as tumor necrosis factor alpha (TNF-α), interleukin-1 beta (IL-1β), and C-X-C motif chemokine ligand 8 (CXCL8), which induce the migration of neutrophils and monocytes—later differentiated into macrophages—toward the site of inflammation [9,10,12,14].

During this process, all signals generated after tissue injury activate G protein–coupled receptors (GPCRs) located on the surface of immune cells. These receptors stimulate adenylate cyclase (AC), a transmembrane enzyme that catalyzes the conversion of adenosine triphosphate (ATP) to cAMP. cAMP functions as an intracellular second messenger, activating regulatory pathways such as those mediated by protein kinase A (PKA) and the transcription factor CREB. These signaling routes modulate the gene expression of cytokines and other inflammation-related proteins, generally exerting an anti-inflammatory effect by suppressing NF-κB activation and other proinflammatory signaling cascades [15,16,17]. In this context, PDE4 plays an essential role by regulating intracellular cAMP levels through its hydrolysis to AMP. Since cAMP acts as a negative modulator of inflammation, PDE4 activity amplifies the inflammatory response by reducing the concentration of cAMP. In contrast, inhibition of PDE4 prevents cAMP degradation, prolonging its signaling activity, and producing an overall anti-inflammatory effect characterized by a decrease in cytokine production (TNF-α, IL-1β, and IL-6) and an increase in IL-10 levels. The release of these mediators, along with others such as leukotriene B4 (LTB4) and histamine, regulates a cascade of events characteristic of the inflammatory response, including vasodilation, increased vascular permeability, upregulation of endothelial adhesion molecules, edema, and recruitment of immune cells [10,14,18].

Inflammation is classified as acute or chronic depending on its duration and intensity. In the acute phase, vascular changes and infiltration of neutrophils and macrophages lead to swelling, pain, and erythema [19]. Once the causal agent is eliminated, the resolution of the process occurs through pro-resolving molecules such as resolvins and lipoxins [20,21]. When resolution is not achieved and the acute inflammatory response persists for an extended period, chronic inflammation is established, which may last for months or even years. In this state, most features of acute inflammation—such as immune cell accumulation and increased vascularization—remain active. However, chronic inflammation is also characterized by enhanced lymphocytic infiltration accompanied by the continuous release of cytokines and reactive oxygen species [19,22]. In both scenarios, inhibition of PDE4 represents a promising therapeutic strategy to attenuate the inflammatory response and promote tissue homeostasis.

### 2.2. Anti-Inflammatory Agents

Currently, the treatment of inflammatory diseases relies primarily on corticosteroids and immunomodulatory agents. Although these compounds are highly effective in reducing inflammation, their long-term use is associated with significant adverse effects, including hypertension, metabolic disturbances, and a progressive loss of therapeutic efficacy over time [23,24]. These limitations have driven the development of phosphodiesterase 4 (PDE4) inhibitors—such as roflumilast, apremilast, and crisaborole—which exert their effects by modulating intracellular cAMP signaling and regulating the production of proinflammatory cytokines without directly compromising overall immune function [25,26]. Nevertheless, the clinical use of these inhibitors is frequently limited by tolerability issues, most notably nausea, headache, and weight loss, which largely arise from their insufficient isoform selectivity.

As a result, current research efforts are focused on the rational design of more selective PDE4 inhibitors and on the exploration of natural compounds or structurally derived analogs capable of establishing more specific interactions with the enzyme, while simultaneously offering an improved safety and tolerability profile [27].

## 3. Phosphodiesterase 4

### 3.1. Enzymatic Role

Phosphodiesterases (PDEs) are present in almost all cell types and are localized within specific subcellular compartments, where they hydrolyze cAMP or cGMP into their non-cyclic forms. Their primary function is to regulate signaling pathways dependent on these cyclic nucleotides, which are generated through the activation of AC or guanylyl cyclase (GC) [28]. Through this mechanism, PDEs control essential physiological processes such as muscle contraction, neurotransmission, inflammatory response, and cell proliferation. Within this enzyme family, PDE4 is distinguished by its cAMP specificity—unlike other PDEs—and by its particularly high abundance in nervous, immune, and respiratory tissues. PDE4 modulates the intensity, duration, and spatial localization of intracellular responses. Its role extends beyond the mere hydrolysis of cAMP to AMP, as it contributes to the organization of the cell’s functional architecture through its specific subcellular distribution, protein–protein interactions, and post-translational regulation [29].

**Figure 1:**
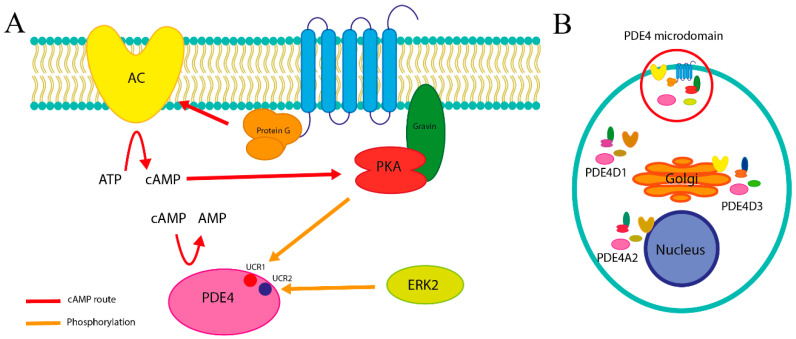
(**A**) Factors controlling cAMP kinetics. Gravin, associated with the plasma membrane, anchors PKA, which regulates activity of PDE4 near the membrane to yield a pattern of cAMP distinct from that seen in other PDE4 microdomains. (**B**) Different microdomains in a hypothetical cell.

This spatial organization, as shown in Figure 1 of intracellular responses, arises from the subcellular localization of PDE4 isoforms, each of which possesses distinct N-terminal sequences that target the enzyme to specific compartments such as the Golgi apparatus, cytoplasm, or nucleus. Within these regions, PDE4 interacts with anchoring proteins such as AKAPs (A-kinase anchoring proteins), gravin, or mAKAP, which bring it into proximity with AC and protein kinase A PKA, responsible for generating cAMP and for continuing the inflammatory cytokine pathway, respectively [30,31]. This arrangement establishes cAMP signaling microdomains, in which the synthesis, diffusion, and degradation of the messenger molecule are tightly controlled. Such compartmentalization ensures highly specific signaling responses and prevents the indiscriminate propagation of cAMP, enabling localized activation of effectors such as PKA that regulate sensitive processes including immune responses and muscle contraction [32,33].

Moreover, PDE4 activity is subject to complex autoregulation through phosphorylation. Its conserved regulatory domains, upstream conserved regions (UCR1 and UCR2), can be phosphorylated by PKA under conditions of elevated cAMP levels, thereby increasing enzymatic activity and establishing a negative feedback loop that restores homeostasis [34,35]. Conversely, phosphorylation mediated by extracellular signal-regulated kinase 2 (ERK2) can inhibit its function when signal attenuation is required [32]. At the transcriptional level, sustained elevation of cAMP also induces PDE4 mRNA expression, reinforcing its role as both a regulatory and organizational enzyme within the signaling microenvironment [36,37]. The finely tuned modulation of PDE4 activity according to the cell’s physiological needs reflects the sophistication of this intracellular control system [17].

### 3.2. Structure of Pde4

The PDE4 enzyme comprises four subfamilies of isoforms (PDE4A–D), each encoded by distinct genes but sharing a highly conserved catalytic domain. Among these isoforms, minor sequence variations lead to subtle structural differences that, in turn, result in differential affinities for inhibitors [38]. Despite these variations, PDE4 enzymes exhibit a highly conserved modular organization consisting of regulatory regions and a central catalytic domain [8,39]. Understanding their overall architecture and its internal dynamics is essential for interpreting the structural differences discussed later, as well as for analyzing the determinants of affinity, selectivity, and geometric compatibility.

**Figure 2:**
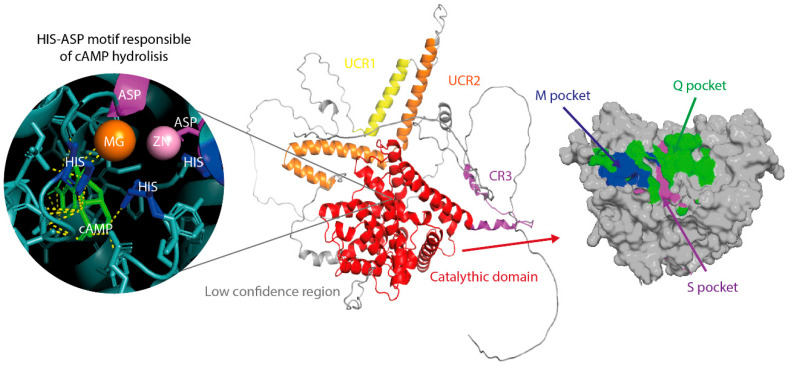
Three-dimensional structure of PDE4B, showing the surface of the different catalytic pockets and the interaction of cAMP with the His–Asp motif.

Each PDE4 isoform consists of three principal regions: an N-terminal regulatory domain, a highly conserved C-terminal catalytic domain, and—present in most long isoforms—an intermediate segment containing the regulatory regions UCR1 and UCR2 (Upstream Conserved Region 1 and 2) as illustrated in Figure 2.

The UCR1 and UCR2 regions participate in autoinhibition, dimerization, and phosphorylation, thereby modulating the accessibility of the catalytic domain. Phosphorylation of UCR1 by protein kinase A (PKA) enhances PDE4 activity by disrupting the inhibitory UCR1–UCR2 conformation. Conversely, phosphorylation of the ERK2 kinase consensus motif (PxS/TP) within the catalytic domain leads to inhibition of the long isoforms, altering the equilibrium between open and closed states and indirectly affecting the geometry of the active site. The functional coupling between UCR1 and CR3 constitutes a “gating” mechanism that regulates substrate and inhibitor access [40,41,42].

The catalytic domain forms the structural and functional core common to all members of the PDE4 family. In all isoforms, this domain has a constant length of approximately 330 amino acids and shares 82–88% sequence identity among subfamilies. Structurally, it adopts a compact α-helical fold composed of 17 α-helices organized into three subdomains that converge to form a deep cavity (~440 Å^3^) housing the catalytic site [43,44].

The PDE4 catalytic site can be functionally described as comprising three interconnected pockets—the Q-pocket, M-pocket, and S-pocket—together with the accessory CR3 domain. These regions exhibit complementary chemical properties that determine substrate and inhibitor selectivity [45]:

**Q-pocket:** This is the main substrate recognition region. It contains an invariant glutamine residue (Gln) responsible for recognizing the adenine base and an aromatic residue (Phe) that establishes a network of hydrogen bonds and π–π stacking interactions with the adenine ring of cAMP or with aromatic inhibitor scaffolds [46]. The pocket includes two hydrophobic subregions, Q1 and Q2, responsible for orienting the nucleotide or ligand within the cavity [44,45].

**M-pocket (metal pocket):** This is the binuclear catalytic center of PDE4, where Zn^2+^ and Mg^2+^ (or Mn^2+^) ions are coordinated and are essential for the hydrolysis of the phosphodiester bond. These metals are arranged in an octahedral geometry through conserved His–Asp motifs. An aspartate bridge links both metal ions at a distance of ~3.9 Å, forming the binuclear catalytic center presented in Figure 2 [43,44].

**S-pocket (solvent pocket):** This corresponds to the most hydrophilic region of the active site. It is composed of polar and charged residues (e.g., glutamates, serines, cysteines, and glutamines) that interact with water molecules, forming a stabilization network for the catalytic complex that helps in efficient binding [44,45].

**CR3 domain:** Located at the entrance to the catalytic site, the CR3 (Capping Region 3) domain acts as a dynamic “lid” that regulates the accessibility of substrates and inhibitors to the catalytic pocket. Although its sequence varies moderately among isoforms, its structural role is conserved across all PDE4s [41,42].

Recent structural models generated by AlphaFold3 (2024) for PDE4A–D confirm the conservation of this overall architecture while revealing that peripheral loops exhibit lower structural confidence scores (pLDDT), suggesting intrinsic flexibility. This flexibility may facilitate adaptation to inhibitors of diverse geometries [44].

### 3.3. Catalytic Function

**Figure 3:**
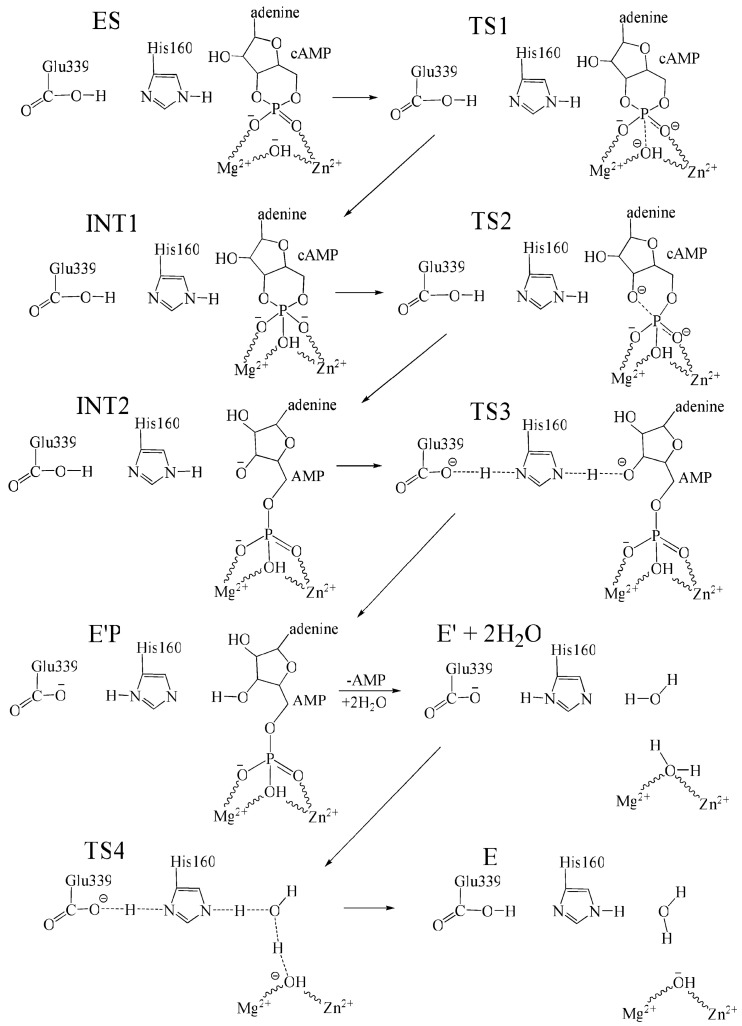
Reaction mechanism for the complete cycle of PDE4-catalyzed hydrolysis of cAMP. For clarity, residues His164, His200, Asp201, Asp 318, Asn321, and Gln859 are hidden from view. Reproduced from [47], with permission from the publisher.

The catalytic mechanism of PDE4 follows a highly ordered and tightly regulated process, as illustrated in Figure 3. The enzyme specifically catalyzes the hydrolysis of cyclic adenosine monophosphate (cAMP) to adenosine monophosphate (AMP). The reaction is initiated when cAMP approaches the active site and enters the catalytic domain through a deep cleft located at its C-terminal region, with the enzyme in an active conformation and the CR3 regulatory “lid” in an open state.

Upon entry, cAMP is positioned within the Q-pocket of the catalytic domain. The nucleotide adopts an orientation that allows its adenine ring to engage in π–π stacking interactions with Phe372 (in PDE4D) and to form hydrogen bonds with Gln369 and Asn321, while the phosphate moiety coordinates with the two divalent metal ions located in the M-pocket. This metal coordination induces polarization of the phosphodiester bond, priming it for cleavage. In this activated configuration, His160 forms a hydrogen bond with the O3′ oxygen of cAMP and is poised to donate a proton, whereas Glu339 functions as a general base, deprotonating His160 and maintaining the catalytic network in equilibrium [43,47,48].

Following stabilization of the enzyme–substrate complex, the bridging hydroxide ion coordinated to the metal ions performs a nucleophilic attack on the phosphorus atom of the cAMP phosphate group. This step leads to the formation of an unstable trigonal–bipyramidal transition-state intermediate, which subsequently collapses. Cleavage of the O3′–P bond results in opening of the phosphodiester ring and conversion of cAMP into AMP. During this process, His160 donates a proton to the departing O3′ oxygen, while Glu339 reprotonates the histidine residue, thereby completing the acid–base catalytic cycle.

The resulting AMP remains transiently coordinated to the metal ions via its phosphate oxygens and is stabilized by electrostatic interactions until it is released from the active site. Product release represents the rate-limiting step of the reaction, with an estimated activation energy of approximately 16 kcal·mol^−1^. Finally, following AMP dissociation, two water molecules enter the catalytic pocket. Through a sequence of proton transfer events mediated by His160 and Glu339, the nucleophilic hydroxide ion required for the subsequent catalytic cycle is regenerated [43,47,48].

### 3.4. Differences Between Isoforms

**Figure 4:**
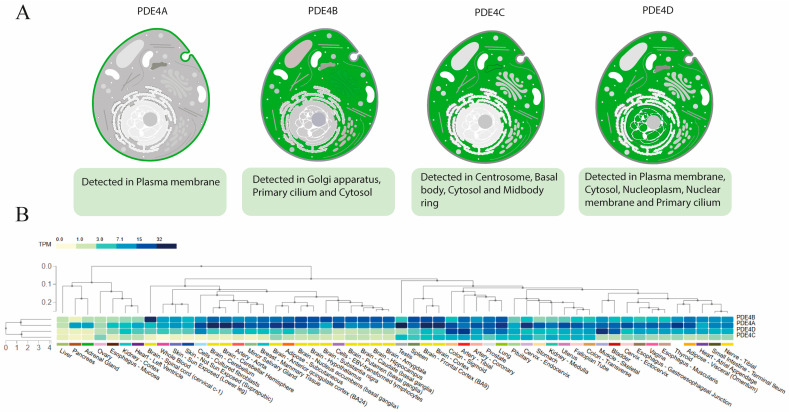
(**A**) Subcellular expression patterns for PDE4 isoforms, obtained from The Human Protein Atlas. Data available at: https://www.proteinatlas.org/ENSG00000184588-PDE4B/subcellular (accessed on 5 November 2025). (**B**) Tissue expression levels of the PDE4 isoforms, obtained through a multigene query in the GTEx Portal (GTEx Consortium), available at https://gtexportal.org/home/multiGeneQueryPage (accessed on 5 November 2025).

The PDE4 enzyme family is composed of four main subfamilies (PDE4A, PDE4B, PDE4C, and PDE4D), each encoded by an independent gene, yet exhibiting an almost identical structural architecture [39]. These different genes exhibit distinct expression patterns across tissues and cell types [49,50]. Analysis of their distribution shows that the various PDE4 isoforms display differential expression both at the tissue level and within immune cell populations. Regarding subcellular localization, the isoforms are found in diverse compartments —including the plasma membrane, cytosol, nucleoplasm, nuclear membrane, Golgi apparatus, centrosome, and primary cilium— indicating that each subfamily operates within specific cellular microdomains, all of this can be seen in Figure 4 [51].

In normal tissues, PDE4B is the isoform with the highest expression levels, showing elevated values in brain, bone marrow, and spleen, whereas PDE4A, PDE4C, and PDE4D exhibit comparatively lower expression across most examined tissues. Among immune cells, the relative expression also varies markedly between isoforms. PDE4B and PDE4D are detected in immune cells, whereas PDE4A and PDE4C show limited or undetectable expression in some of these cell types [52]. Under inflammatory conditions, only PDE4B appears upregulated following lipopolysaccharide (LPS) stimulation in mice, and similarly, its expression is elevated in peripheral blood mononuclear cells (PBMCs) from psoriatic patients, a pattern not observed for PDE4A, PDE4C, or PDE4D [5,18,53]. With respect to kinetic parameters, the isoforms also differ in their interaction with cAMP. PDE4D exhibits the highest substrate specificity, as reflected by its lower Km (1.5 ± 0.1 μM) and relatively high Kcat (5.4 ± 1.0 s^−1^), resulting in a higher catalytic efficiency (Kcat/Km = 3.5 ± 0.7 s^−1^ μM^−1^) compared with PDE4A, PDE4B, and PDE4C [54].

The canonical forms of these subfamilies possess a catalytic domain that reflects significant functional conservation within the active site [44,54]. However, the multiple exons that compose the four PDE4 subfamilies undergo alternative splicing, generating a wide diversity of isoforms. These isoforms form distinct isoform-selective cellular microdomains and participate in the regulation of PDE4 activity through interactions with accessory proteins [30,31,55]. As a result, they exhibit different expression levels and molecular functions across various tissues and cell types [56,57,58].

**Figure 5:**
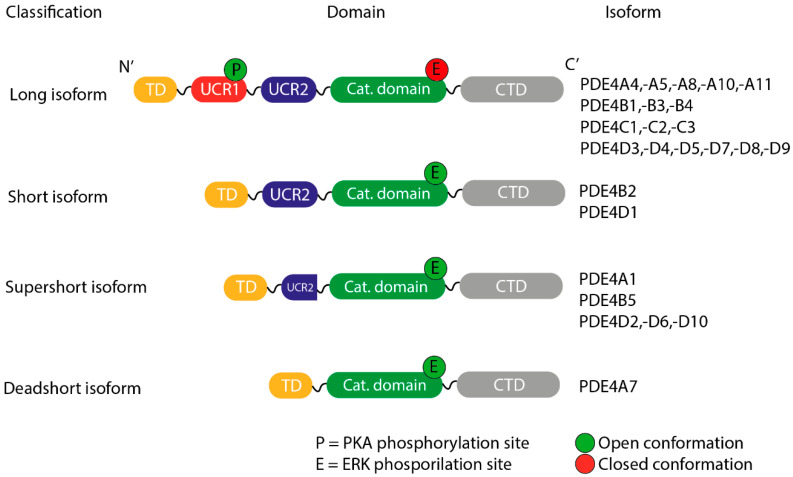
PDE4 isoform structure and classification. This is an adapted version of figures in a previous report [61] and has been modified. The isoforms are classified according to the components resulting from their processing, which are: TD, transduction domain; UCR, upstream conserved region; CTD, C-terminal domain.

All PDE4 subfamilies possess an upstream conserved region (UCR) responsible for determining the enzyme’s state and controlling intracellular signaling. According to the presence or absence of these regulatory regions, the isoforms are classified into four groups which are clearly depicted in Figure 5 [59,60,61]:

- Long, containing both UCR1 and UCR2.
- Short, containing only UCR2.
- Super-short, featuring a truncated version of UCR2.
- Ultra-short (dead-short), which completely lacks these regions.

The importance of the UCR modules lies in their role in the functional regulation of PDE4. This regulation occurs mainly through phosphorylation by protein kinase A (PKA) and extracellular signal-regulated kinase (ERK). UCR1 serves as a phosphorylation site for PKA, increasing the activity of long isoforms and allowing precise spatial control of intracellular cAMP levels. In contrast, ERK-mediated phosphorylation differentially affects the isoforms: it inhibits the long forms, activates the short ones, and does not modify the super-short variants [62,63,64,65].

**Figure 6:**
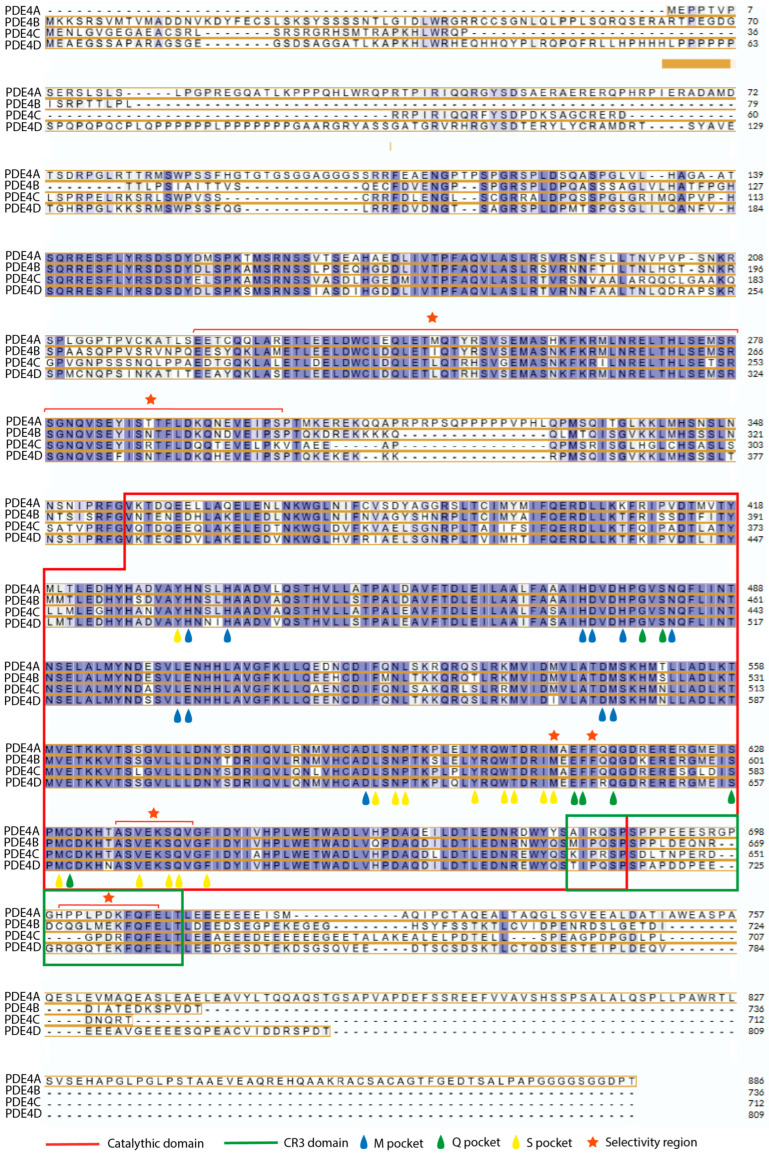
Sequence alignment of the 4 isoforms, indicating the catalytic domain, the CR3 domain, the residues belonging to the different pockets, and the selectivity regions, redrawn and modified from the concept presented in a previous report [44], sequence alignment of the PDE4 catalytic domains was performed using the Clustal Omega 1.2.4 program [69], in the UniProt Knowledgebase (UniProtKB) Release 2025_04, protein sequences corresponding to human PDE4A (UniProt ID: P27815), PDE4B (Q07343), PDE4C (Q08493), and PDE4D (Q08499) were retrieved from UniProt.

Therefore, each isoform exerts a slightly different modulatory function within the cell, fine-tuning the intensity and duration of cAMP-mediated signaling in specific physiological contexts. Although these functions are not yet fully understood, the study of structural differences among isoforms provides essential information about their functional and pharmacological selectivity. As shown in the alignment of the PDE4 sequence in Figure 6, the active site is highly conserved between subfamilies, with 254 of the 325 residues (78%) being identical [54]. This conservation is clearly visible in the invariant residues highlighted in the alignment, particularly those involved in metal coordination, the Q-switch, and the P-clamp pocket so in this zone the selectivity comes from subtle changes, also visible in the alignment, that correspond to structural features previously described in the literature that can modulate inhibitor affinity. In contrast, regions outside the catalytic center show substantially lower conservation, and it is within these variable segments where clear isoform-specific differences emerge.

PDE4B and PDE4D are the most structurally similar, representing the main challenge in achieving isoform selectivity. However, there are some distinctions, such as the Met583 residue in PDE4B, which connects the helices H14 and H15 and exhibits marked conformational differences compared to its equivalent Met639 in PDE4D [54]. In addition, changes in amino acids located in the CR3 region lead to greater local flexibility that may influence inhibitor binding [66]. In particular, a single residue substitution—Leu674 in PDE4B versus Gln730 in PDE4D—alters the conformation of the C-terminal helix and its coupling with ligands that produce high selectivity for inhibitors [38]. However, the most prominent difference is found outside the catalytic center in the UCR2 domain; this low conservation sequence modulates interactions with allosteric inhibitors, specifically, a change from Tyr274 in PDE4B to Phe332 in PDE4D generates high specificity in the development of inhibitors [67,68].

In PDE4A, the structural superposition with PDE4D reveals a greater divergence than that observed between PDE4B and PDE4D. PDE4A shows a significant displacement of N-terminal residues adjacent to the invariant glutamine (Ala635–Ser641/Gln642), which may be a key determinant of its selectivity [54].

In PDE4C, the differences are more pronounced. Helices H8 and H9 of the H-loop, as well as parts of H14 and H15, appear disordered or lack a defined electron density, and the residues of the H and M-loops—critical for inhibitor interaction—also exhibit structural disorder. In addition, the side chain of Phe568 adopts a different orientation compared to that observed in PDE4D [54]. These variations make the catalytic site of PDE4C the most distinctive among the PDE4 subfamilies.

## 4. Natural Bioactives as Pde4 Inhibitors

PDE4 inhibitors represent one of the most widely employed strategies for the treatment of inflammatory diseases, as well as other pathological conditions. However, its clinical development has been consistently limited by an unfavorable tolerability profile. The main dose-limiting adverse effects (nausea, vomiting, diarrhea, and headache) are closely linked to systemic PDE4 inhibition and, in particular, to increased cAMP in central regions involved in the emetic response, such as the area postrema, with a significant contribution from the PDE4D isoform [70]. This narrow therapeutic margin has been the main reason for abandoning the development of numerous synthetic PDE4 inhibitors administered orally, despite their proven pharmacodynamic efficacy [71]. Therefore, the fundamental problem with this pharmacological class is not a lack of efficacy, but the impossibility of achieving sufficient inhibition of inflammation without inducing systemic toxicity. In this context, research into natural PDE4 inhibitors is of particular interest, as these compounds tend to have moderate potency and partial or less selective inhibition of isoforms associated with central adverse effects, which could translate into a better balance between anti-inflammatory efficacy and tolerability. Although synthetic inhibitors such as roflumilast, apremilast, and crisaborole are currently used, the discovery of natural compounds with inhibitory capacity has opened a new avenue for rational drug design characterized by lower complexity and reduced toxicity.

### 4.1. Approved Inhibitors

PDE4 inhibitors constitute one of the most extensively investigated therapeutic strategies for the treatment of multiple inflammatory disorders, including asthma, COPD, psoriasis, atopic dermatitis, rheumatoid arthritis, inflammatory bowel disease, lupus and various conditions [6,25,26,72]. The development of these agents has progressed from first-generation inhibitors such as rolipram, which exhibited notable potential in neurological pathologies but whose significant adverse effects—including nausea, vomiting, and headache—limited its clinical application [73]. Building upon this compound, a second generation emerged, exemplified by roflumilast, the first PDE4 inhibitor approved for the treatment of COPD and subsequently asthma. Roflumilast demonstrated superior efficacy and an improved safety profile compared with rolipram, although it remains associated with gastrointestinal adverse effects such as diarrhea, nausea, weight loss, and headache [74,75,76]. In parallel, other inhibitors of this generation, such as cilomilast, showed anti-inflammatory and bronchodilatory activity in preclinical models and clinical trials, as well as high biochemical specificity, inhibiting PDE4B and PDE4D in the nanomolar range while exhibiting minimal activity against other PDE families; however, they ultimately failed to achieve clinical approval [7,77].

More recently, a third generation of PDE4 inhibitors has emerged, among which apremilast stands out. Approved in 2014 for plaque psoriasis and psoriatic arthritis, its clinical applications have since expanded to include Behçet’s disease, cicatricial alopecias, and cutaneous lupus, although adverse effects such as headache, abdominal pain, diarrhea, nausea, vomiting, and weight loss persist [78,79,80]. Crisaborole, approved in 2016 for atopic dermatitis, represents a significant advance in topical formulations, avoiding the systemic toxicity characteristic of oral inhibitors and exhibiting a favorable pharmacokinetic profile with rapid conversion to inactive metabolites [81,82].

Despite these ongoing improvements, adverse effects remain a major challenge, continuing to drive the search for inhibitors with greater isoform selectivity, improved tolerability, and more refined pharmacological profiles. For this reason, the use of natural compounds as inhibitory pharmacophores has gained prominence, as they offer the potential to overcome these limitations. The relevance of these compounds lies not only in their biological origin but also in the fact that they possess privileged structural scaffolds that reproduce the geometric features of the natural substrate and exhibit intrinsic activity toward the catalytic pockets. Moreover, due to their electron-rich distribution of hydrogen-bond donors and acceptors, they adapt more effectively to the polar microenvironment of the active site. In addition, their low basal toxicity, acceptable bioavailability, and chemical versatility make them suitable for semi-synthetic modification, leading to derivatives with enhanced potency or selectivity for specific PDE4 isoforms [83].

### 4.2. Natural Compounds with Inhibition

As shown in Table 1, there is a wide variety of natural compounds capable of inhibiting PDE4 activity. All these structures share common features, such as the presence of conjugated aromatic systems that promote π–π interactions with residue Phe372 (in PDE4D); hydrogen-bond donor or acceptor groups that enable hydrogen bonding with critical polar residues of the catalytic site; and hydrophobic regions that fit into the apolar zones of the catalytic pocket, increasing affinity [84,85,86]. These characteristics allow them to partially mimic the adenine base of cAMP, resulting in competitive inhibition against the natural substrate and preventing the entry of water required for hydrolysis.

Among the inhibitors listed in Table 1, several compounds have been evaluated across different PDE4 isoforms, revealing distinct subtype selectivity profiles. For example, baicalin exhibits an IC_50_ value of 4.8 μM against PDE4B, while showing markedly reduced activity toward PDE4D [87]. A similar trend has been reported for mesembrenone from *Sceletium tortuosum* and for the millesianins isolated from *Millettia dielsiana*. In contrast, other compounds, such as moracin M, display higher affinity for PDE4D (IC_50_ = 2.9 μM) compared to PDE4B (IC_50_ = 4.5 μM) [88,89,90]. These examples highlight that natural PDE4 inhibitors can exhibit measurable and sometimes pronounced isoform selectivity, underscoring their potential as starting points for subtype-selective inhibitor development.

**Table 1:** Natural compounds that exhibit inhibition activity against PDE4. * For this compound, no IC_50_ value has been reported in the literature, although another piece of information regarding its inhibition has been documented.

Name	Structure	Taxonomy	Plant	IC50	Conditions	Isoform	References
Braylin	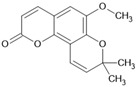	Coumarin	*Hypericum sampsonii*	1.27 μM	Radiometric assay	PDE4D	[91]
Toddacoumalone	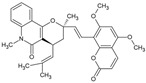	Coumarin	*Toddalia asiatica*	0.14 μM	Radiometric assay	PDE4D	[92,93]
Cyclomorusin	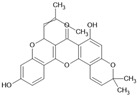	Flavonoid	*Morus alba*	0.0054 μM	Radiometric assay	PDE4D	[94]
Polycyclic polyprenylated acylphloroglucinols	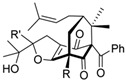	Terpenoid	*Hypericum sampsonii*	0.64 μM	Radiometric assay	PDE4D	[95]
Pentacyclic triterpene G1	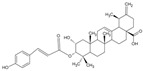	Terpenoid	*Gaultheria yunnanensis*	0.245 μM	Radiometric assay	PDE4D	[85]
Alkaloid mesembrenone	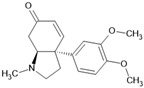	Alkaloid	*Sceletium tortuosum*	0.47 μM	Radiometric assay	PDE4B	[89]
Forsythin	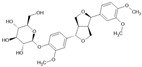	Lignan	*Forsythia suspensa*	1.8 μM	Fluorescence-based KIT	PDE4D	[96]
Psidial A	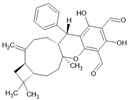	Terpenoid	*Psidium guajava*	1.6 μM	Radiometric assay	PDE4D	[97]
Selagintamarlin A	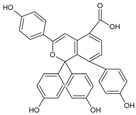	Phenolic compound	*Selaginella tamariscina*	0.049 μM	TR-FRET	PDE4D	[98]
Moracin M	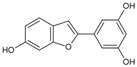	Phenolic compound	*Morus alba*	2.9 μM	Radiometric assay	PDE4D	[88]
Resveratrol	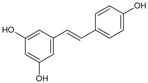	Phenolic compound	*Vitis vinifera*	18.8 μM	Radiometric assay	PDE4D	[99,100]
Amentoflavone	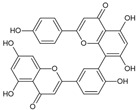	Flavonoid	*Platycladus orientalis*	* 74.2% at 0.2 µg/mL	Radiometric assay	PDE4D	[101]
Sinigrin	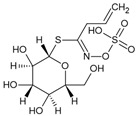	Glucosinolatos	*Brassica*	Nd	Nd	Nd	[102]
Osthol	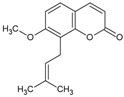	Coumarin	*Angelica hirsutiflora*	7.81 nM	Oxidative stress in activated neutrophils	Nd	[103]
Baicalin	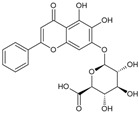	Flavonoid	*Pourthiaea villosa*	4.8 μM	TR-FRET	PDE4B	[87]
Curcumin	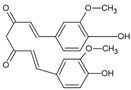	Polifenol	*Curcuma longa*	19.82 μM	Radiometric assay	Nd	[104]
Dioclein	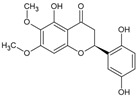	Flavonoid	*Dioclea grandiflora*	16.8 μM	Radiometric assay	Nd	[105]
Millesianins	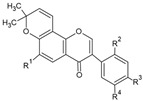	Flavonoid	*Millettia dielsiana*	6.56 μM	Fluorescence-based KIT	PDE4B	[90]

### 4.3. Rational Design Based on Natural Inhibitors

Structural studies of PDE4 isoforms have revealed conformational differences among the isoforms, particularly in regions such as CR3. These differences generate isoform-dependent sub-pockets adjacent to the conserved catalytic site, which can be selectively exploited for inhibitor design. This strategy enables the development of inhibitors with up to 100-fold selectivity for PDE4B and can be applied to natural scaffolds.

Natural PDE4 inhibitors exhibit remarkable structural diversity. Some compounds, such as selagintamarlin A or toddacoumalone, display complex polycyclic architectures and potent affinity for PDE4; however, their modification and large-scale production are difficult. In contrast, within the family of natural PDE4 inhibitors, there are simpler structures that offer versatile chemical scaffolds, allowing the introduction of targeted substitutions or hybridization with other pharmacophoric motifs to optimize both affinity and selectivity toward specific PDE4 isoforms. Therefore, although the exploration of complex natural products serves as a valuable source of potent inhibitors, rational design strategies assisted by molecular modeling can yield natural compound–derived inhibitors with enhanced activity and selectivity.

There are already studies in which derivatives with strong inhibitory capacity have been developed from resveratrol [100]; however, the most interesting advances come from structure-based derivatives, such as curcumin-derived inhibitor **4e** *4,4′-((1E,1′E)-(1-(4-methoxyphenyl)-1H-pyrazole-3,5-diyl)bis(ethene-2,1-diyl))bis(2-methoxyphenol)*. Here, a partial cyclization of the central skeleton of curcumin was designed to generate a heterocyclic pyrazole ring, to which a p-methoxyphenyl aromatic ring was subsequently attached through the nitrogen atom of the heterocycle. These structural modifications conferred greater rigidity and planarity to the molecule, enabling stronger π–π stacking interactions with the hydrophobic residues Phe372, Phe340, and Ile336, as well as hydrogen bonds with Gln433 and Met357 within the catalytic site. In addition, the additional methoxy group facilitated new hydrophobic interactions with His160 and His204, contributing to the stabilization of the enzyme–inhibitor complex, ultimately resulting in an IC_50_ value of 0.023 µM [104].

Additionally, α-Mangostin was selected as an initial scaffold for the design of PDE4 inhibitors after identifying key structural similarities with classical inhibitors such as rolipram and roflumilast, particularly the presence of a catechol motif capable of interacting with conserved residues within the catalytic site. Based on previously reported crystallographic structures of PDE4–inhibitor complexes, it was predicted that α-mangostin could establish hydrophobic interactions with the so-called hydrophobic clamp of the enzyme; therefore, the introduction of targeted modifications was expected to enable exploitation of additional regions of the active site. Guided by this hypothesis, cyclized derivatives were rationally designed to increase molecular rigidity and planarity, thereby favoring π–π stacking interactions, and substituents were subsequently introduced at strategic positions to reach the metal-coordination region. These structural modifications were evaluated using molecular modeling and docking approaches, which confirmed that derivative **4e***((5-Hydroxy-8-methoxy-2,2-dimethyl-7-(3-methylbut-2-en-1-yl)-6-oxo-2H,6H-pyrano[3,2-b]xanthen-9-yl)oxy)heptanoic Acid* adopts an unexpected binding mode in which the carboxyl group extends toward the metal-binding zone of the active site and directly coordinates the Zn^2+^ ion, forming multiple hydrogen bonds with key residues such as His160 and His164. Through this structure-based analysis and rational modification, a high-affinity PDE4 inhibitor was obtained, with an IC_50_ value of 17 nM [106].

### 4.4. Future Perspectives

After analyzing the current progress in the search for PDE4 inhibitors capable of controlling inflammation without adverse effects, it is necessary to consider what lies ahead.

Currently, several trends are being explored in the search for PDE4 inhibitors. In addition to classical competitive inhibitors, alternative approaches such as allosteric modulation and the use of proteolysis-targeting chimeras (PROTACs) represent some of the most recent developments in this field. Allosteric inhibition, particularly targeting regulatory regions such as UCR2 or CR3, is increasingly considered a complementary and promising strategy for modulating PDE4 activity [107]. In contrast, PROTAC-based approaches remain scarcely explored for PDE4 and are strongly influenced by multiple factors that still raise significant uncertainties, including isoform dependence, cellular permeability, and the risk of undesired protein degradation [108]. In this context, competitive inhibition—the focus of the present review—remains the most advanced and reliable strategy, providing a higher level of confidence for the rational identification and optimization of effective PDE4 inhibitors.

One of the main goals is clearly the design or discovery of isoform-selective inhibitors to avoid the side effects associated with PDE4D. There is already evidence of target regions capable of distinguishing between isoforms, and studies have successfully designed natural compound derivatives with improved inhibition based on the structural features of the enzyme. Focusing on the isoform-selectivity hotspots and broadening our perspective by exploring specificity sites outside the catalytic center could lead to the development of compounds that fulfill these objectives. Another promising direction is the search for natural compounds that inherently possess these selectivity characteristics. Although more challenging, the vast structural diversity of secondary metabolites ensures that some natural molecules will likely exhibit isoform-selective binding to distinct PDE4 subtypes.

To achieve this goal, it is highly useful to rely both on literature reporting experimentally validated interactions between PDE4 and known inhibitors, and on specialized structural databases. Among these, PDEStrIAn stands out as a platform that systematically compiles and analyzes more than two hundred crystallographic structures of PDEs, including PDE4, their bound ligands, and the subpockets of the catalytic site [109]. This database is capable of classifying interactions by well-defined residues within their respective pockets and distinguishing structural features among different isoforms, enabling the identification of interaction patterns and differential binding determinants that can be exploited for the design of isoform-selective inhibitors. In this way, PDEStrIAn becomes a valuable tool both for rational drug design and for the identification of natural candidates with interaction profiles compatible with the desired selectivity.

Furthermore, with the recent advances in computational chemistry, artificial intelligence (AI) and machine learning (ML) have emerged as key tools to accelerate this process. Deep neural network–based molecular prediction models enable the screening of large databases of natural and synthetic compounds to identify those most likely to interact with critical residues within the catalytic site. Generative AI platforms such as DeepChem, Schrödinger’s Deep AutoQSAR, ChemBERTa, and AlphaFold-Multimer, when combined with docking and molecular dynamics simulations, can provide optimized compounds and rational structural modifications with improved fit and binding affinity. Altogether, these integrated computational and experimental approaches define the most promising path forward in the pursuit of next-generation PDE4 inhibitors.

## 5. Conclusions and Outlook

PDE4 represents a crucial therapeutic target for the treatment of inflammatory diseases due to its central role in cAMP degradation and, consequently, in the regulation of multiple cellular signaling pathways. In recent years, advances in structural and mechanistic studies have provided detailed insights into the organization of the PDE4 catalytic domain and the conformational differences among its isoforms, thereby enabling the rational design of more selective inhibitors with improved pharmacological profiles.

By integrating key structural features of the studied molecules—such as aromatic rings and metal-binding groups that mimic the enzyme’s natural substrate, along with molecular flexibility and rotational freedom that facilitate optimal accommodation within the catalytic pocket and interactions with hydrophobic residues—with the known structural similarities and differences among PDE4 isoforms, it becomes possible to predict and identify promising molecular frameworks for isoform-specific PDE4 inhibition.

Within this context, natural compounds emerge as particularly attractive candidates owing to their structural resemblance to endogenous substrates, low toxicity, and cost-effective development. Furthermore, natural product–based scaffolds support a sustainable drug discovery paradigm, as they are derived from renewable biological sources and often exhibit broad pharmacological potential. Natural scaffolds such as curcumin and α-mangostin have already undergone targeted chemical modifications at key catalytic residues, yielding inhibitors with enhanced potency and isoform specificity.

Finally, future efforts should focus on integrating recent computational advances with emerging experimental approaches to further identify and optimize isoform-specific PDE4 inhibitors with minimal toxicity. The combined application of structure-based drug design, molecular dynamics simulations, and targeted chemical modifications is expected to accelerate the development of next-generation PDE4 inhibitors, ultimately enabling more precise modulation of inflammatory processes and improved therapeutic outcomes.

## Data Availability

No new data were created or analyzed in this study.

## Abbreviations

PDE4: Phosphodiesterase 4; PDEs: Phosphodiesterases; cAMP: Cyclic adenosine monophosphate; AMP: Adenosine monophosphate; cGMP: Cyclic guanosine monophosphate; GMP: Guanosine monophosphate; ATP: Adenosine triphosphate; TLRs: Toll-like receptors; PRRs: Pattern recognition receptors; TNF-α: Tumor necrosis factor alpha; IL-1β: Interleukin-1 beta; IL-6: Interleukin-6; IL-10: Interleukin-10; CXCL8: C-X-C motif chemokine ligand 8 (interleukin-8); GPCRs: G protein–coupled receptors; AC: Adenylate cyclase; GC: Guanylyl cyclase; PKA: Protein kinase A; CREB: cAMP response element-binding protein; NF-κB: Nuclear factor kappa-light-chain-enhancer of activated B cells; LTB4: Leukotriene B4; AKAPs: A-kinase anchoring proteins; mAKAP: Muscle-specific A-kinase anchoring protein; ERK2: Extracellular signal-regulated kinase 2; UCR1: Upstream conserved region 1; UCR2: Upstream conserved region 2; CR3: Control region 3; COPD: Chronic obstructive pulmonary disease.
